# P-1879. Journal Club Revitalization Initiative at an Academic Infectious Disease Fellowship Program from 2024-2025

**DOI:** 10.1093/ofid/ofaf695.2048

**Published:** 2026-01-11

**Authors:** Alex Belote, Erica MacKenzie, Albert Eid, Charles Walde, Rachel Sigler

**Affiliations:** University of Kansas Medical Center, Kansas City, Kansas; University of Kansas Health System, Kansas City, Kansas; University of Kansas Medical Center, Kansas City, Kansas; University of Kansas Medical Center, Kansas City, Kansas; University of Kansas Medical Center, Kansas City, Kansas

## Abstract

**Background:**

Journal Club (JC) has served as an integral part of medical education for over 150 years. JC was introduced at the University of Kansas Medical Center (KUMC) in 1962 and has evolved over the years. An initiative to improve JC quality, variety and participation was performed, including alternative formats. The aim of this study was to assess revitalization initiatives introduced for JC in 2024.

**Figure:**
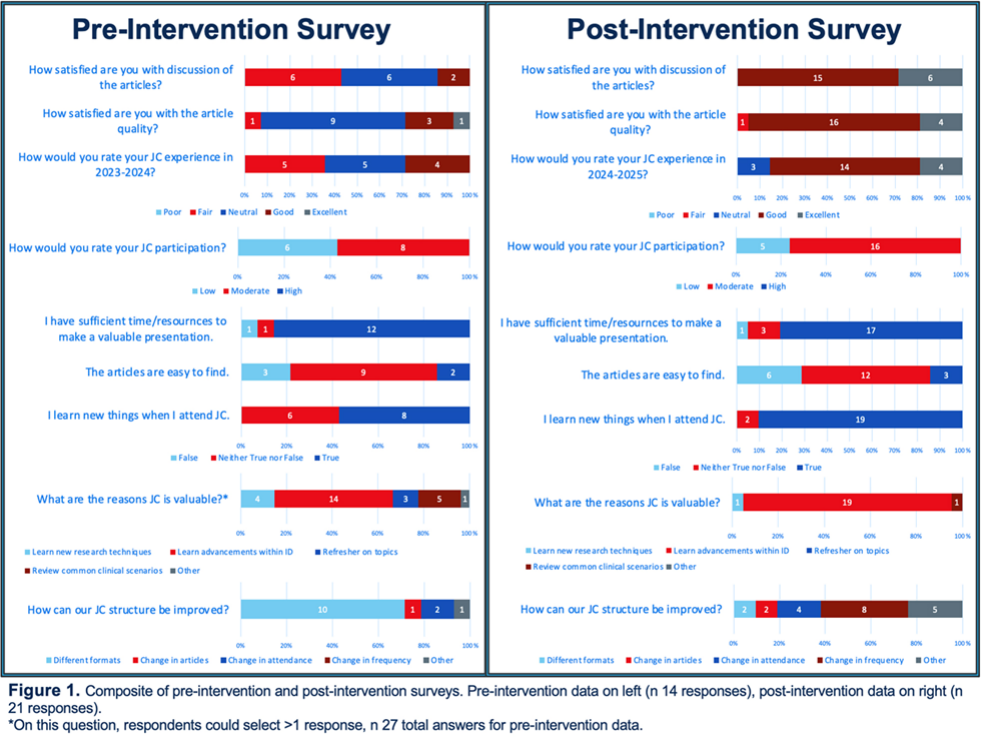

**Figure:**
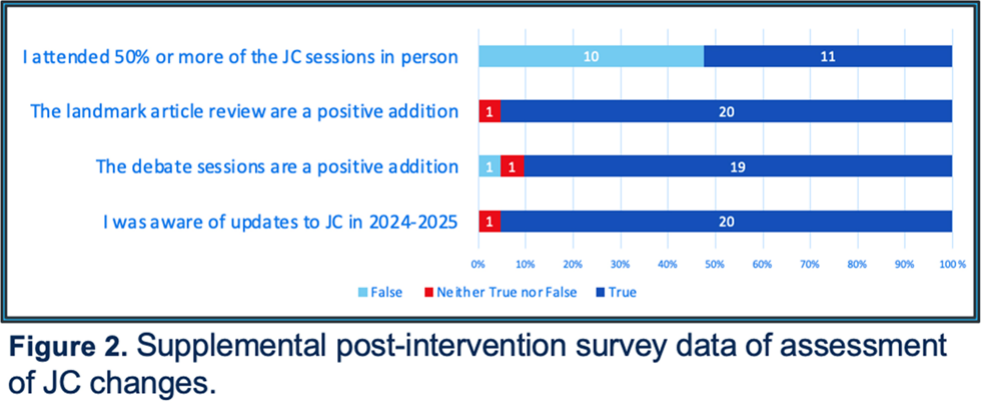

**Methods:**

We distributed a pre-intervention 9-question satisfaction survey in April 2024 to faculty and fellows within our Infectious Disease (ID) division. A ‘journal club restructure’ committee elected to adopt alternative formats, including ‘Landmark’, ‘Debate’, and ‘Deep-dive’ sessions. Following these measures in 2024-2025, a 13-question post-intervention survey was distributed, including the original 9 questions, with an additional 4 questions aimed at assessing the interventions.

**Figure:**
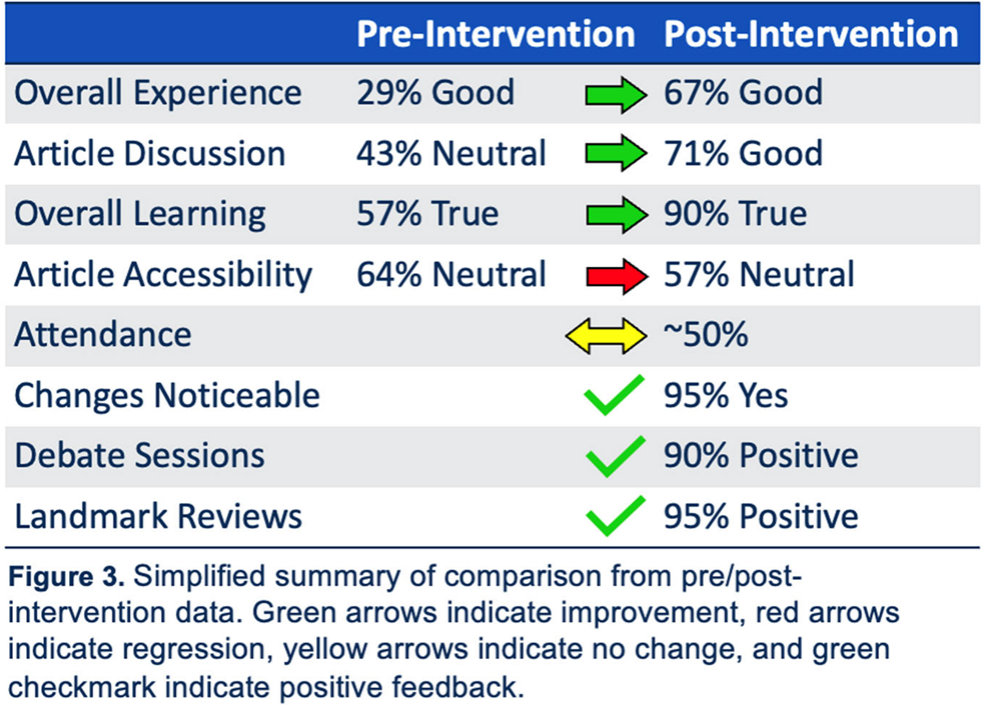

**Results:**

There were 14 pre-intervention survey responses. JC experiences were mixed between fair, neutral and good. Self-rated participation in JC was low and moderate. Most respondents (71%) felt the best way to improve JC was to utilize different formats.

There were 21 post-intervention survey responses. A vast majority of respondents (95%) had improved satisfaction with JC. Most felt the addition of debate and landmark article sessions were a positive addition (90% and 95%, respectively). In-person attendance did not change. Satisfaction with article quality and discussion improved. Ongoing feedback suggestions included reduction in frequency, increased in-person attendance, and presentation of higher impact articles.

**Conclusion:**

Our Infectious Disease division implemented changes to JC based on faculty/fellow feedback. Addition of debate and landmark article sessions were both well-received, with over 90% of respondents noting them as valuable additions. There were several notable opportunities for ongoing improvement, including participation and frequency of JC. This change resulted in significant improvement in JC satisfaction.

**Disclosures:**

All Authors: No reported disclosures

